# Restoration of disk height through non-surgical spinal decompression is associated with decreased discogenic low back pain: a retrospective cohort study

**DOI:** 10.1186/1471-2474-11-155

**Published:** 2010-07-08

**Authors:** Christian C Apfel, Ozlem S Cakmakkaya, William Martin, Charlotte Richmond, Alex Macario, Elizabeth George, Maximilian Schaefer, Joseph V Pergolizzi

**Affiliations:** 1 Perioperative Clinical Research Core, Department of Anesthesia and Perioperative Care, University of California San Francisco, San Francisco, California, USA; 2 Upper Valley Interventional Radiology. McAllen, Texas, USA; 3 NEMA Research, Inc, Biomedical Research & Education Foundation, LLC, Miami Beach, FL, USA; 4 Departments of Anesthesia and Health Research and Policy, Stanford University, Palo Alto, California, USA; 5 Department of Medicine, Johns Hopkins University, Baltimore, Maryland, & Department of Anesthesia, Georgetown University School of Medicine, Washington, DC, USA

## Abstract

**Background:**

Because previous studies have suggested that motorized non-surgical spinal decompression can reduce chronic low back pain (LBP) due to disc degeneration (discogenic low back pain) and disc herniation, it has accordingly been hypothesized that the reduction of pressure on affected discs will facilitate their regeneration. The goal of this study was to determine if changes in LBP, as measured on a verbal rating scale, before and after a 6-week treatment period with non-surgical spinal decompression, correlate with changes in lumbar disc height, as measured on computed tomography (CT) scans.

**Methods:**

A retrospective cohort study of adults with chronic LBP attributed to disc herniation and/or discogenic LBP who underwent a 6-week treatment protocol of motorized non-surgical spinal decompression via the DRX9000 with CT scans before and after treatment. The main outcomes were changes in pain as measured on a verbal rating scale from 0 to 10 during a flexion-extension range of motion evaluation and changes in disc height as measured on CT scans. Paired t-test or linear regression was used as appropriate with p < 0.05 considered to be statistically significant.

**Results:**

We identified 30 patients with lumbar disc herniation with an average age of 65 years, body mass index of 29 kg/m^2^, 21 females and 9 males, and an average duration of LBP of 12.5 weeks. During treatment, low back pain decreased from 6.2 (SD 2.2) to 1.6 (2.3, p < 0.001) and disc height increased from 7.5 (1.7) mm to 8.8 (1.7) mm (p < 0.001). Increase in disc height and reduction in pain were significantly correlated (r = 0.36, p = 0.044).

**Conclusions:**

Non-surgical spinal decompression was associated with a reduction in pain and an increase in disc height. The correlation of these variables suggests that pain reduction may be mediated, at least in part, through a restoration of disc height. A randomized controlled trial is needed to confirm these promising results.

**Clinical trial registration number:**

NCT00828880

## Background

An estimated 80% of the population will suffer from low back pain (LBP) at some point of their lives[1]. Low back pain is the number one factor limiting activity in patients less that 45 years old, the second most frequent reason for doctor's visits, and the third most common cause for surgical procedures[2]. In addition to imposing upon patients' quality of life, LBP is of significant socioeconomic relevance because it may lead to a temporary loss of productivity, enormous medical and indirect costs, or even permanent disability[3].

While the management of persistent low back pain remains hotly debated, the traditional approach has been non-surgical treatment with analgesia supplemented by physiotherapy. Given the limited efficacy of these modalities, there are also a number of alternative interventions such as massage, spinal manipulation, exercises, acupuncture, back school and cognitive behavioral therapy[4]. The two most common diseases involving chronic LBP are discogenic low back pain, responsible for 39% of cases, and disc herniation, accounting for just less than 30% of LBP incidence. These incidence frequencies are supported by the current data that most closely link the clinical pathology of discogenic low back pain and disc herniation to the anatomical structure of the intervertebral disc. Thus, another treatment option is motorized decompression, a technique designed to lessen pressure on the discs, vertically expand the intervertebral space, and restore disc height[5-7]. However, systematic reviews to date were unable to find sufficient evidence in the literature to support the use of this modality[8,9]. A subsequent chart review of 94 patients suggests that motorized non-surgical spinal decompression may be effective in reducing chronic low back pain[10]. Furthermore, preliminary data from a prospective cohort study in patients with chronic low back pain reported a median pain score reduction from 7 to 0 (on a 11-point verbal rating scale) following a 6-week non-surgical spinal decompression treatment protocol[11].

The goal of this study was therefore to determine if changes in LBP, as measured on a verbal rating scale, before and after a 6-week treatment period with motorized non-surgical spinal decompression, correlate with changes in lumbar disc height, as measured on computed tomography scans.

## Methods

### Study design

This is a retrospective cohort study of patients who underwent a 6-week treatment protocol of non-surgical spinal decompression via the DRX9000. A HIPAA (Health Insurance Portability and Accountability Act) waiver was obtained through Quorum IRB. This waiver permitted a review of medical records and access to CT scans ordered as part of standard of care.

Clinical Trial Registration Number: NCT00828880

### Inclusion and exclusion criteria

Patients and their medical records were eligible for inclusion if the patient was at least 18 years of age, consented for the 6-week treatment protocol, and presented with chronic LBP of at least 3 out of 10 on a verbal rating scale and was due to either discogenic LBP or disc herniation according to a radiological diagnosis using standard medical definitions. Discogenic LBP is most succinctly defined as a loss of lower back function with pain due to disc degeneration. Degenerative disc diseases often emerge when abnormal stresses cause the nucleus gelatinosus to unevenly distribute weight, the annular fibrosis and end plate incur structural damage, and a destructive inflammatory response is triggered to accelerate and perpetuate the degeneration of the disc. A herniated disc (synonymous with a protruding or bulging disc) arises when the intervertebral disc degenerates and is weakened to such an extent that cartilage is pushed into the space containing the spinal cord or a nerve root and causes pain[1].

All patients were treated at the Upper Valley Interventional Radiology facility (McAllen, Texas). Patient symptoms were evaluated by medical history review, physical examination, and a current CT scan (not older than 2 months prior to the start of treatment) to support a diagnosis of chronic discogenic LBP due to bulging, protruding or herniated intervertebral discs that may have been brought on by degenerative disc disease. Patients were only included if pre- and post-treatment CT scans were performed on the same device, measurements taken by the same investigator (WM), and data recorded on standard collection forms. One height measurement was taken by WM for each of the intervertebral discs under study per CT scan. Accuracy of data was confirmed by a second investigator (JP), but only one measurement was made of each intervertebral disc per CT scan. All CT scans analyzed were performed at least one hour after the subject got out of bed. The first CT scan was performed within two months before the initiation of the treatment, and the second CT scan at least one day after or on the day immediately before the final treatment session.

Exclusion criteria for enrollment in the study were any patients with metastatic cancer; previous spinal fusion or placement of stabilization hardware, instrumentation or artificial discs; neurologic motor deficits; bladder or sexual dysfunction; alcohol or drug abuse; or litigation for a health-related claim (in process or pending for workers' compensation or personal injury). Limitations of the spinal decompression system also led to the exclusion of patients with extremes of height (< 147 cm or > 203 cm) and body weight (> 136 kg).

### Treatment protocol

Patients received treatment with the DRX9000 (Axiom Worldwide, Tampa, FL) as dictated by the intervention's operating guidelines[11]. In short, the protocol typically included 22 sessions of spinal decompression over a 6-week period with 28-minute active treatment sessions. At the start of each session, the patient is fitted with adjustable lower and upper body harnesses and is lowered into the supine position. To initiate active treatment the machine then pulls the patient gently on the lower harness while the upper harness remains stationary, thus distracting the patient's spine. A safety button can be pushed at any time by the patient to release all tension immediately. Daily treatments, Monday through Friday, were performed for the first two weeks of treatment. The latter four weeks consisted of treatments every other day, Monday, Wednesday and Friday.

Initial decompression force was adjusted to patient tolerance, starting at 4.54 kg (10 lbs) less than half their body weight. If a patient described the decompression pull as "strong or painful," this distraction force was decreased by 10%-25%. In subsequent treatment sessions, the distraction force was increased as tolerated to final levels of 4.54 kg to 9.07 kg (10 to 20 lbs) more than half their body weight. Patients continued to use analgesics prescribed by their physicians before enrollment, but were allowed to use additional non-steroidal pain medication should their pain increase temporarily and permitted to discontinue pain medication as needed. During the routine physical examination performed by WM prior to beginning the non-surgical spinal decompression treatment session, at the first and final visits maximal pain was evaluated during a flexion-extension range of motion exam with the question "How strong is your pain on a scale of 0-10 with 0 being no pain and 10 as bad as it could be?"

### Variables

The first main outcome for this study was the change in pain during a range of motion evaluation measured on an 11-point verbal rating scale (VRS), with 0 being no pain and 10 being pain as excruciating as could be imagined, before and after the 6-week spinal decompression treatment regimen.

The second main outcome was the change in average disc height as measured by CT scan. For each patient, average disc height of L3-L4, L4-L5 and L5-S1 was calculated before the first treatment session and at least one day after or on the day before the last treatment session.

### Statistical analysis and sample size estimation

We assumed data to be normally distributed unless exploratory analyses suggested otherwise, in which case a Kolmogorov-Smirnov test was to be applied. Since the treatment effect was defined as the difference between before and after the therapeutic intervention, a paired t-test was applied to test whether there was a reduction in pain and an increase in disc height. For the main hypothesis, the correlation between disc height changes and low back pain, we applied linear regression to quantify the relationship with Pearson's correlation coefficient to determine statistical significance.

Sample size estimations were performed to have sufficient power to test with a two sided type I error of 0.05 and type II error of 0.2 (80% power). Given the sizeable treatment effect reported in the retrospective chart review and also in the prospective pilot study mentioned in the introduction, we expected a reduction in range of motion pain from 6 to 2, with a standard deviation of 2.5. This resulted in a sample size estimation of only 5 patients. To test changes in disc height, we expected a standard disc height of about 8 mm with diseased discs being slightly more compressed, i.e. at about 7.5 mm, and anticipated discs after the decompression treatment to measure at about 8.25 mm. Assuming a standard deviation of 1.0 mm, we estimated a required sample size of 16 patients in order to show a difference. The sample size for the main hypothesis, that the degree of pain reduction is associated with the amount of increase in disc height, was more difficult to estimate since no previous study had determined a correlation coefficient. Therefore, we chose a coefficient of 0.5 for a conservative expectation, resulting in a required sample size of 26 patients. Taking into consideration the possibility of drop-outs, we aimed to collect data from 30 patients.

## Results

During a two year period, Sept 19, 2005 to Aug 6, 2007, a total of 103 patients were treated with the intervention, but only 30 of those patients fulfilled the per protocol inclusion and exclusion criteria for the analysis. The 30 participants consisted of 21 female and 9 male patients with lumbar disc herniation. They had a mean (SD) age of 65 (± 15) years, a body mass index of 29 (± 5) kg/m^2^, and an average duration of LBP of 12.5 (± 19) weeks with a score of 6.3 (± 2.2) on the VRS (Table 1). All 30 patients had a disc prolapse and the majority (n = 25) also had degenerative disc disease.

**Table 1:** Patient characteristics

Patient characteristics:	Mean (±SD)
Age (yr)	64.4 (±14.9)
Height (cm)	166.1 (±8.5)
Weight (kg)	80.5 (±14.4)
BMI (kg/m^2^)	28.8 (±5.0)
Gender (F/M)	70% (21/9)
Average disk height, pre-treatment (mm)	7.5 (±1.7)

**Pain:**	

Pain, palpation (before first visit, 0-10)	6.2 (±2.2)
Pain, range of motion (before first visit, 0-10)	6.2 (±2.2)
Pain duration (weeks)	12.5 (±19.4)

**Diagnosis:**	

Herniation (simple)	5
Herniation (with degenerative disk disease)	25

**Disk Levels (with corresponding traction angles):**	

L3-L4 & L4-L5 (15-20°)	1
L4-L5 (15°)	11
L4-L5 & L5-S1 (10-15°)	6
L5-S1 (10°)	12

The maximum force during the first treatment was on average 33.9 (± 6.8) kg and gradually increased during subsequent treatment visits to 52.4 (± 7.6) kg (Table 2).

**Table 2:** Treatment characteristics and outcome

	First Visit	Last Visit	Change (SD); p-value
Maximal traction force (kg)	33.9 (±6.8)	52.4 (±7.7)	
Pain, palpation (0-10)	6.2 (±2.2)	1.6 (±2.3)	-4.5 (±2.7), <0.001
Pain, range of motion (0-10)	6.2 (±2.2)	1.6 (±2.3)	-4.5 (±2.7), <0.001
Average disk height (mm)	7.5 (±1.7)	8.8 (±1.7)	1.3 (±0.5), <0.001

**Figure 1:**
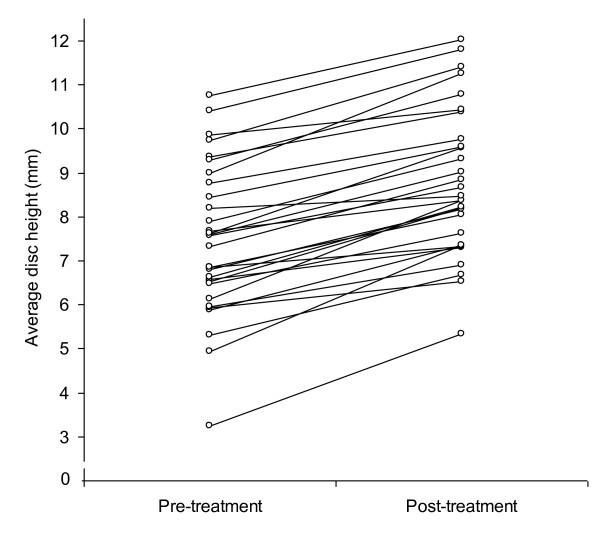
**Increase in disk height before and after the non-invasive spinal decompression treatment protocol**.

**Figure 2:**
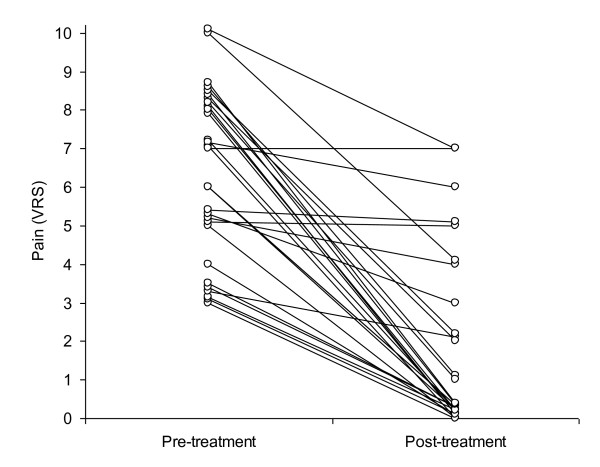
**Pain reduction before and after the non-invasive spinal decompression treatment protocol (because several lines overlap, there are less lines than subjects)**.

Low back pain decreased from 6.2 (± 2.2) to 1.6 (± 2.3, p < 0.001) and disc height increased from 7.5 (± 1.7) to 8.8 (± 1.7) mm (p < 0.001) (Figures 1 and 2).

**Figure 3:**
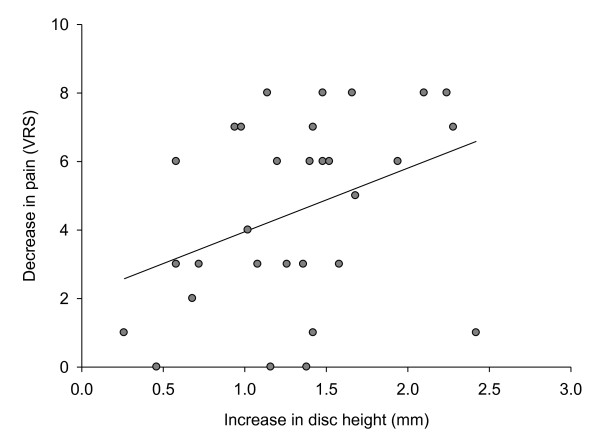
**Correlation between increase in disk height and decrease in pain**.

There was a statistically significant correlation between the increase in disc height and a reduction in pain (r = 0.36, p = 0.044), with a 1 mm increase in disc height being associated with a reduction of 1.86 on the 11-point verbal rating scale (Fig. 3). No adverse events were reported during the treatment period.

## Discussion

In this cohort study we extracted data from 30 patients with discogenic low back pain and found an average reduction in pain from 6.2 to 1.6 after non-surgical spinal decompression. This level of pain relief is consistent with two previous studies using DRX9000 to decrease chronic low back pain[10,11]. However, here we systematically investigated the change in disc height before and after the treatment, and were able to show that increases in disc height correlated with increased pain relief. A mechanical explanation for this correlation might be that the non-spinal decompression reduces the pressure on the discs. This relief of stress would simultaneously promote regeneration of diseased and compressed discs and increase lumbar disc height, with the latter reducing load on the facet joints.

It is well recognized that continuous pressure on vertebral discs decreases their height. Humans are taller in the morning after the discs decompress while the body is supine overnight and shorter in the evening after the discs have borne weight during daily activity[12]. Interestingly, this effect occurs quite rapidly so that the majority of height-loss in a day occurs within the first hour of arising. Therefore, all CT scans analyzed in this study were performed at least one hour after the subject got out of bed. The first CT scan was performed within two months before the initiation of the treatment and at least one day after or the day immediately before the final treatment session.

A clear diagnosis cannot be made in approximately 80% of cases of LBP, and imaging techniques can only offer a partial solution to the problem of making a causal diagnosis of LBP[13]. One might argue that a CT scan is not as sensitive a measure of disc height as an MRI scan because it images soft tissues poorly and cannot examine internal disc morphology. However, because the primary objective was to establish an observable correlation between disc height increase and decreased LBP, a CT scan permitting examination of the outline of the intervertebral discs at high resolution provided sufficient measurable evidence[14].

It has been demonstrated that low back pain can lead to muscle spasms that could directly perpetuate pain,[15] or induce pain within the disc as nerve fibers have been described to grow into the inner part of the annulus fibrosus or nucleus pulposus[16]. It is hypothesized that the pain-spasm-pain cycle[15] is perpetuated by further reduction in disc height, which also simultaneously aggravates the facet joint. In either case, dampened pressure on the disc should facilitate the regeneration of the disc and assuage facet joint stress. In fact, it has been described that non-surgical spinal decompression mechanically creates negative intradiscal pressures, and it is speculated that this supports disc regeneration, though this remains controversial[5].

Pain measurement relies first and foremost on patient report. Taking into account the subjectivity inherent in this process, it was noted that a cut-off point, or rather the change in pain score necessary for detecting a clinically important difference in an individual patient, was needed to identify responders and non-responders to analgesia. Farrar et al reported that on average a reduction in pain intensity of at least 2 points on the NRS serves as a clinically significant change[17]. Using this standard, in this cohort study this intervention had a success rate of over 75% (pain decreased by more than 2 out of 11 in 23 out of 30 patients). In our analysis, each millimeter of increase in disc height was associated with pain relief of roughly 2 points on the scale, a clinically important difference according to the aforementioned report.

However, not all patients responded equally. This raises the question of inter-individual variability and might be addressed by taking into account the heterogeneity of lumbar spine muscle strength acting as a counterforce to the external distraction. Even though the DRX9000 machine has an integrated sensor to detect counterforces, non-surgical spinal decompression can only work if lumbar spine muscles are relaxed. Another reason for different inter-individual response rates could be the age of the patients. However, in sub-analyses (not described) we did not find a correlation between age and treatment success. With regards to the elderly cohort of patients analyzed in this retrospective study, it is possible that a younger patient population might respond differently to the non-surgical spinal decompression treatment given that they would generally have less disc degeneration, be more active, and have less co-morbidity than the elderly population studied here. Yet this is a hypothesis that remains to be tested in a future prospective study investigating therapies to alleviate LBP in younger patients. While we largely believe the range of muscle tone during non-surgical spinal decompression to be the main reason for different treatment effects, other reasons for variability could be differing stages and degrees of degenerative disc disease, an assortment of activity levels, and a wide spectrum of concomitant treatments ranging from chiropractic interventions and pain medication cocktails.

One limitation of this study is the lack of a control group. This is especially relevant for herniated discs, because of the significant rate of spontaneous recovery[18,19]. A control group would have been absolutely necessary if the primary objective was to establish a causal relationship proving that the increase in disc height is due to the non-surgical spinal decompression; however, our primary objective was rather to demonstrate the correlation between increased disc height and reduction of pain. Thus, irrespective of a control group, this is the first study that provides evidence of an association between an anatomical correlate, change in disc height, with pain relief over time. Even so, it is possible the placebo effect may have contributed to the perception of having decreased pain. Given that the correlation between the increase of disc height and the reduction of pain shows an r^2 ^= .13, while statistically significant, there is room for an argument suggesting that perhaps the placebo effect played a role in the positive outcome. Both limitations of the current retrospective study indicate the need for a randomized placebo-controlled trial to establish a more concrete relationship between the anatomical disc changes attributed to the non-surgical spinal decompression intervention and the reduction of LBP.

Patients with chronic discogenic low back pain are usually on a wide range of analgesics, and pain and analgesic consumption is generally positively correlated. As a result, interventions that reduce pain typically lead to a reduced consumption of analgesics and thus counteract the treatment effect of the intervention (suppressor effect). The fact that a significant reduction of pain was observed even though analgesics were not controlled for corroborates the observation of pain relief through non-surgical spinal decompression.

Finally, the follow-up period was too short to comment on the permanency of pain relief. However, this was not within the scope of this study and the duration of the effect is not essential to substantiate our primary finding that restoration of disc height through non-surgical spinal decompression is associated with decreased discogenic low-back pain. The next step will be to obtain long-term results, e.g. 1 or 2 years after the last treatment cycle, to a) investigate whether treatment effects are long lasting and to b) more importantly, establish whether there is a long term correlation between disc height increase and pain reduction.

## Conclusions

In this study of non-surgical spinal decompression for chronic discogenic low back pain we were able to demonstrate an association between the restoration of disc height and pain relief. The correlation of these variables suggests that pain reduction may be mediated, at least in part, through a restoration of disc height. These results call for a randomized placebo-controlled trial to substantiate the efficacy and elucidate the mechanism of this promising treatment modality.

## Competing interests

The authors themselves declare that they have no competing interests.

NEMA Research is a Clinical Research Organization that is involved in evidence-based research development and was the lead sponsor implementing the protocol for this clinical trial on behalf of Axiom-Worldwide.

## Authors' contributions

CA contributed to the statistical analysis and drafting the manuscript, OSC contributed to the statistical analysis of the data, WM is responsible for the assessments made, data collection, and data review, CR performed statistical analysis and assisted with writing the manuscript, AM assisted with drafting the manuscript, EG contributed to drafting, editing, and formatting the manuscript, MS contributed to drafting and editing the manuscript, JVP performed the data review. All authors read and approved the final manuscript.

## Pre-publication history

The pre-publication history for this paper can be accessed here:

http://www.biomedcentral.com/1471-2474/11/155/prepub
